# Flagellar arrangements in elongated peritrichous bacteria: bundle formation and swimming properties

**DOI:** 10.1140/epje/s10189-021-00027-8

**Published:** 2021-03-08

**Authors:** Judit Clopés, Roland G. Winkler

**Affiliations:** 1 Theoretical Physics of Living Matter, Institute of Biological Information Processing and Institute for Advanced Simulation, Forschungszentrum Jülich and JARA, 52425 Jülich, Germany; 2 Institute for Theoretical Physics, RWTH Aachen University, 52074 Aachen, Germany

## Abstract

**Abstract:**

The surface distribution of flagella in peritrichous bacterial cells has been traditionally assumed to be random. Recently, the presence of a regular grid-like pattern of basal bodies has been suggested. Experimentally, the manipulation of the anchoring points of flagella in the cell membrane is difficult, and thus, elucidation of the consequences of a particular pattern on bacterial locomotion is challenging. We analyze the bundle formation process and swimming properties of *Bacillus subtilis*-like cells considering random, helical, and ring-like arrangements of flagella by means of mesoscale hydrodynamics simulations. Helical and ring patterns preferentially yield configurations with a single bundle, whereas configurations with no clear bundles are most likely for random anchoring. For any type of pattern, there is an almost equally low probability to form V-shaped bundle configurations with at least two bundles. Variation of the flagellum length yields a clear preference for a single major bundle in helical and ring patterns as soon as the flagellum length exceeds the body length. The average swimming speed of cells with a single or two bundles is rather similar, and approximately $50\%$ larger than that of cells of other types of flagellar organization. Considering the various anchoring patterns, rings yield the smallest average swimming speed independent of the type of bundle, followed by helical arrangements, and largest speeds are observed for random anchoring. Hence, a regular pattern provides no advantage in terms of swimming speed compared to random anchoring of flagella, but yields more likely single-bundle configurations.

**Graphic abstract:**

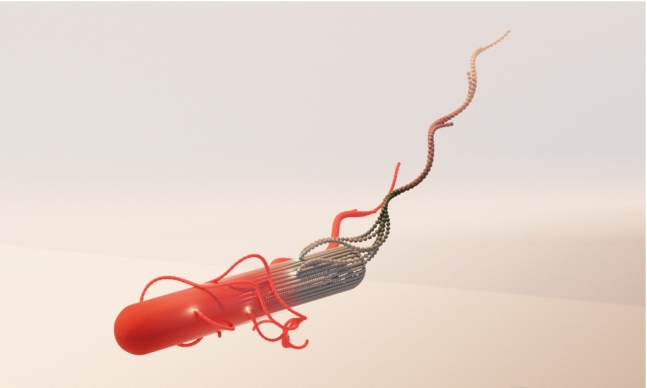

## Introduction

The majority of motile bacterial species are propelled by flagella [1], which protrude from their cell body and are driven by rotary motors [2–4]. The number of flagella and their arrangement differs widely among bacterial species, ranging from a single flagellum (monotrichous bacteria) to many flagella distributed all over the body (peritrichous bacteria) [5–7]. Well-studied examples of peritrichous bacteria are *Escherichia coli*, *Bacillus subtilis*, *Salmonella enterica*, and *Proteus mirabilis*. The biological advantage in some species to produce more than one flagellum is still not clear, and might have more implications apart from improving the swimming motility [8, 9]. Interestingly, the cell size and number of flagella of peritrichous bacteria can depend on their mode of locomotion. Individual (planktonic) cells exhibit the so-called swimming motility [2, 6, 10–12], where the various flagella self-organize into bundles by (typically) counterclockwise rotation of the motors. Surface-associated bacteria display another mode of motility denoted as swarming, where they migrate collectively over surfaces while forming stable, highly motile aggregates [6, 10–15]. Swarming bacteria show a strikingly different motile behavior than swimming cells. They are densely packed and exhibit large-scale turbulent-like streaming motions [13]. Various bacteria undergo substantial morphological changes while transiting from a planktonic to a swarmer cell, as they become more elongated by suppression of cell division and their number of flagella significantly increases [5, 6, 11, 16–18]. So far, it is unclear why swarming requires multiple flagella or significantly elongated cells [6, 8, 9]. Even more, the interactions between the large number of flagella within and between swarmer cells are unresolved, although experiments suggest that interwoven flagellar bundles between neighboring cells can be formed [16, 17]. This would require formation of several flagellar bundles within a cell, which should point away from the cell body.

A major unresolved issue is the arrangement of the flagella on a peritrichous cell’s surface, and whether this plays a role in its propulsion. Despite the large number of flagellated bacteria [1], only a few flagellar surface distribution patterns seem to exist [19]. The distribution of flagella on the body surface of peritrichous bacteria has been traditionally assumed to be random [6, 20, 21], although some studies suggest an organized surface arrangement, for instance, in positions inherited from those of the ancestor cell in *E. coli* [22], or in a grid-like fashion in *B. subtilis* [7].

Studies on *B. subtilis*, a non-pathogenic Gram-positive bacterium species living in ubiquitous environments ranging from soils and plant roots to the gastrointestinal tract of animals [23], reveal the existence of several flagellar bundles [24, 25]. Vegetative *B. subtilis* cells are capable to switch from the planktonic state in a three-dimensional fluid to a swarming phenotype and form a biofilm [14, 15]. Flagella growing from basal bodies, whose positions remain fixed in both the planktonic and the swarming state, power active movement in both locomotion modes [21, 26]. Contrary to *P. mirabilis* or *E. coli*, *B. subtilis* cells show minor differences in body length between swimming and swarming cells [8]. Due to their ability to adapt to a wide range of environments, the genome of different strains of *B. subtilis* shows a large diversity [23] and has been subsequently classified into subspecies due to the presence of strand-specific genes [27].

In this article, we present mesoscale hydrodynamics simulation results of *B. subtilis*-like cells to illustrate the role of flagellar arrangement patterns on the formation of flagellar bundles and the bacterium swimming properties. Flagellar arrangement has received little attention so far, but insight into pattern-specific properties may help to understand not only locomotion of individual cells, but may also shed light onto the collective behavior of swarmers, specifically possible inter-cell flagellum–flagellum interactions due to particular bundle arrangements.

We employ a mechano-elastic non-tumbling bacterium model that accounts for near-field hydrodynamics, i.e., provides a length-scale resolution smaller than the bacterium diameter [3, 28–30]. The embedding fluid is modeled by the multiparticle collision dynamics (MPC) approach, a particle-based mesoscale fluid model which captures hydrodynamic interactions and thermal fluctuations [31, 32]. Fluid-mediated interactions have been proven to be fundamental for flagellum-flagellum synchronization [33–37] and bundle formation [37, 38] in microswimmers. MPC has been successfully employed to simulate cell swimming [3, 28–30, 39, 40], the synchronization of flagella in sperm [41] and in cilia arrays [42], and bundle formation in bacteria [3, 37, 38].

Our simulations reveal a strong influence of the anchoring pattern on the formation of bundles. Specifically ring patterns (grid) yield a clear preference for single bundles, whereas random anchoring often results in configurations without well-defined bundles or peculiarly swimming cells. The swimming speed is higher for single- and two-bundle configurations, independent of the anchoring pattern. Remarkably, random anchoring yields the largest average swimming speed, independent of the number of formed bundles. Hence, our simulations reveal no advantage of a regular anchoring pattern in terms of swimming speed compared to random anchoring.

The paper is structured as follows. In Sect. 2, the bacterium and the MPC fluid model are presented. Section 3 provides results for the obtained flagellar organizations of the various anchoring patterns. In Sect. 4, the cell dynamics is discussed. Finally, Sect. 5 provides conclusions and a summary of our findings.

**Fig. 1:**
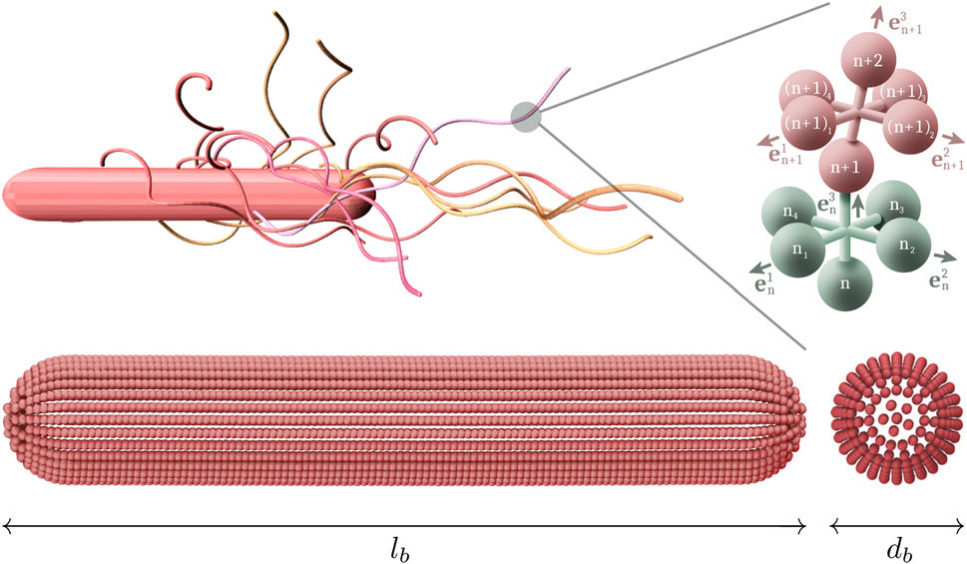
Model of a bacterial cell with 14 flagella anchored randomly on the cell body. The body consists of a cylinder with spherical caps, and a flagellum is composed of overlapping octahedral units

## Bacterium simulation approach

### Bacterium model

The mechano-elastic bacterium model of Refs. [3, 28–30], adapted to *B. subtilis*-like cells, is applied, where a cell is composed of a spherocylindrical body and attached semiflexible helical filaments (Fig. 1). The whole cell is constructed by discrete points (beads) of mass *M* connected by harmonic springs with the bond potential

1 $$\begin{aligned} U_b = \frac{1}{2}K_b(r-l_0)^2 , \end{aligned}$$

where *r* is the distance between a respective bead pair, $l_0$ the rest length, and $K_b$ the spring constant.

#### Model of the body

The body is comprised of rings of 30 beads arranged on the circumference of a circle of diameter $d_b$, with a ring spacing of 0.5*a* (along the cylinder center line)—*a* is the length unit related to the MPC fluid described in Sect. 2.2—and a bead in the center of the circle. Each of the spherical caps consists of 9 rings of decreasing diameter, with 4 rings toward the pole with the smaller bead numbers 15, 15, 5, and 1, respectively. The cylindrical shape is maintained by strong bonds along the ring circumference between next, next-nearest, the $5\mathrm{th}$, $10\mathrm{th}$, $15\mathrm{th}$, $20\mathrm{th}$, and $25\mathrm{th}$ bead, a bond with the central particle, as well as bonds to the next and next-nearest beads of neighboring rings. The length of the body, $l_b$, is adjusted via the number of rings in the cylindrical part.

#### Flagellum model

Each flagellum is modeled as a helical wormlike chain [43–45] adjusted for a suitable implementation in a MPC fluid [28]. A flagellum is composed of $N_s$ overlapping octahedron-like segments, where each isolated segment consists of 6 beads linked by 12 bonds along the edges with rest length $l_0=a/\sqrt{2}$, and three bonds along the diagonals with $l_0=a$ (Fig. 1). In a flagellum, backbone beads of subsequent segments are fused into one to form a linear chain. This model is suitable for describing the intrinsic twist of the helix backbone and facilitates the coupling to the forces exerted by the MPC fluid and vice versa [28]. The $n\mathrm{th}$ segment can be characterized by the backbone bond-vector $\varvec{b}_n^3= \varvec{r}_{n+1}-\varvec{r}_{n}$ ($n=1,\dots ,N_s$), with $\varvec{r}_n$ the position of the backbone particle *n*, and the (nearly) orthogonal vectors $\varvec{b}_n^1= \varvec{r}_{n_1}-\varvec{r}_{n_3}$ and $\varvec{b}_n^2= \varvec{r}_{n_2}-\varvec{r}_{n_4}$ (Fig. 1). Orthonormal triads $\{\varvec{e}_n^1, \varvec{e}_n^2, \varvec{e}_n^3\}$ are introduced as $\varvec{e}_n^{\gamma }=\varvec{b}_n^{\gamma }/| \varvec{b}_n^{\gamma }|$ ($\gamma \in \{1,2,3 \}$). The transport of the triad $\{\varvec{e}_n^1, \varvec{e}_n^2, \varvec{e}_n^3\}$ to the consecutive segment $\{\varvec{e}_{n+1}^1, \varvec{e}_{n+1}^2, \varvec{e}_{n+1}^3\}$ defines the local elastic deformation of the flagellum [43, 46]. This transport consists of both a rotation of the plane formed by $\varvec{e}_n^1$ and $\varvec{e}_n^2$ around $\varvec{e}_n^3$ by a twist angle $\phi _n$ and a rotation of this twisted triad by a bending angle $\theta _n$ around the unit vector $\varvec{n}_n=\big (\varvec{e}_n^3 \times \varvec{e}_{n+1}^3 \big )/|\varvec{e}_{n}^3 \times \varvec{e}_{n+1}^3|$. The harmonic elastic deformation energy $U_{\text {el}}$ of the helical wormlike backbone chain [43–45] is then given by

2 $$\begin{aligned} U_{\text {el}} = \frac{1}{2} \sum _{\alpha =1}^3 K^{\alpha }_{\text {el}} \sum _{n=1}^{N_s-1} \big ( \varOmega _n^{\alpha } - \varOmega _e^{\alpha } \big ), \end{aligned}$$

where $K_{\text {el}}^1=K_{\text {el}}^2$ characterize the bending energy, $K_{\text {el}}^3$ the twist energy, and $\varvec{\varOmega }_n=\sum _{\alpha }\varOmega _n^{\alpha } \varvec{e}_n^{\alpha }=\theta _n \varvec{n}_n + \phi _n \varvec{e}_n^3$ is the strain vector. The parameters $\varOmega _e^{\alpha }$ define the equilibrium geometry of the model flagellum and are chosen to recover the shape of a left-handed flagellum in the normal state [47]. Counterclockwise rotation of a flagellum is achieved by a motor torque $\varvec{T}$ decomposed in a force couple $\varvec{F}$ and $\varvec{-F}$ applied to the particles $1_2$ and $1_4$ of the first triad. As a consequence, the bacterium is force free. An opposite torque $\varvec{-T}$ is applied to the body to ensure that the bacterium is also torque free. The hook is not described explicitly in this model [48]. Each flagellum is directly anchored at the body by choosing a body particle as its first contour particle ($n=1$). The anchoring position of each flagellum to the body is determined according to the chosen pattern and will be described in more detail in Sect. 2.4. Steric interactions between flagella and between a flagellum and the cell body are captured by the purely repulsive harmonic potential

3 $$\begin{aligned} U_{\text {ex}}^{\text {f/b}} = \frac{K_{\text {ex}}}{2} \big (r^{\text {f/b}}-r_{\text {ex}}^{\text {f/b}} \big )^2 \end{aligned}$$

for $r^\text {f/b}<r_{\text {ex}}^\text {f/b}$, and $U_{\text {ex}}^{\text {f/b}} = 0$ for $r^\text {f/b} \ge r_{\text {ex}}^\text {f/b}$. The superscript $\text {f}$ and $\text {b}$ refer to flagellum–flagellum and flagellum–body interactions, respectively. $K_{\text {ex}}$ is the strength of the repulsive potential, $r_\mathrm{ex}^\text {f}$ the closest distance between two contour bond segments of different flagella [49], and $r_\mathrm{ex}^\text {b}$ that between a flagellum segment and the body-center line. We set $r_{\text {ex}}^\mathrm{f}=0.25a$ and $r_{\text {ex}}^{b}=(d_b+a)/2$.

### Fluid model: multiparticle collision dynamics (MPC)

In MPC, the fluid is represented by point particles of mass *m*, whose dynamics proceeds in alternating steps—streaming and collision—updating the particle positions $\varvec{r}_i$ and velocities $\varvec{v}_i$ ($i=1,\ldots , N$), respectively [31, 32, 50]. In the streaming step, the MPC fluid particles move ballistically during a collision time *h* and their positions are updated as

4 $$\begin{aligned} \varvec{r}_i(t+h) = \varvec{r}_i(t) + h\varvec{v}_i(t) . \end{aligned}$$

Coupling and momentum exchange between particles takes place in the collision step. Here, the particles are sorted into the cells of a cubic lattice of mesh size *a*, which defines the local interaction environment. The velocities of the fluid particles after the collision in a cell, $\varvec{v}_i(t+h)$, are given by [51, 52]

5 $$\begin{aligned} \varvec{v}_i(t+h) =&\ \varvec{v}_{\text {cm}}(t) + \mathbf{R} (\alpha ) \varvec{v}_{i,\text {cm}}(t) - \varvec{r}_{i,\text {cm}} \nonumber \\&\times \!\! \Bigg [ m \mathbf{I} ^{-1} \sum _{j\in \text {cell}} \varvec{r}_{j, \text {cm}} \!\times \! (\varvec{v}_{j, \text {cm}}-\mathbf{R} (\alpha )\varvec{v}_{j, \text {cm}}) \Bigg ], \end{aligned}$$

within the angular-momentum-conserving stochastic-rotation-dynamics variant of MPC (MPC-SRD+a) [51, 52]. In this equation, $\mathbf{R} (\alpha )$ represents the rotation matrix of the relative velocity of the $i\mathrm{th}$ particle, $\varvec{v}_{i,\text {cm}}(t+h) = \varvec{v}_i(t+h)-\varvec{v}_{\text {cm}}(t+h)$, after streaming with respect to the center-of-mass velocity of the cell, $\varvec{v}_{\text {cm}}$, by a fixed angle $\alpha$ around a randomly oriented axis [53]. The orientation of the axis is chosen independently for every cell and collision step. $\mathbf{I}$ is the moment-of-inertia tensor of the particles in the cell center-of-mass reference frame, and $\varvec{r}_{i, \text {cm}}(t+h)=\varvec{r}_{i}(t+h) - \varvec{r}_{\text {cm}}(t+h)$ is the position of the $i\mathrm{th}$ particle with respect to the center-of-mass position, $\varvec{r}_{\text {cm}}$, of the particles in the cell. Discretization of space in collision cells breaks Galilean invariance, which is restored by a random shift of the collision lattice at every collision step [54]. In order to maintain a constant temperature, the cell-level Maxwell–Boltzmann-scaling canonical thermostat (MBS) is applied [53]. The MPC algorithm is highly parallel, therefore we employ a graphics processor unit (GPU)-based version for a high performance gain [55].

#### Coupling the bacterium and MPC fluid

The coupling of the bacterium and the MPC fluid particles is established in the MPC collision step, where bacterial beads are treated similar to MPC particles and their velocities are also rotated according to Eq. (5). The center-of-mass velocity of a collision cell is then

6 $$\begin{aligned} \varvec{v}_\text {cm} = \frac{1}{mN_c + M N_c^c} \left( \sum _{i=1}^{N_c}m\varvec{v}_i + \sum _{k=1}^{N_c^c}M \varvec{v}_k^c \right) , \end{aligned}$$

where $N_c$ is the number of MPC particles, $N_c^c$ the number of bacterial cell beads in the particular collision cell, and $\varvec{v}_k^c$ is the velocity of a bacterial bead. With this coupling, we ensure the momentum transfer between the bacterial body, flagella, and the fluid. Fluid particles freely penetrate the body surface. However, the collisional coupling suffices to drag the fluid particles inside the body along when it moves. We even satisfactorily achieve no-slip boundary conditions by this coupling as demonstrated for a sedimenting spherical colloid [56].

### Parameters

Simulation parameters The three elastic constants are chosen as $K_\mathrm{el}^1=K_\mathrm{el}^2=K_\mathrm{el}^3=5\times 10^4\ k_\mathrm{B}T$, where $k_\mathrm{B}$ is the Boltzmann constant and *T* the temperature. This yields a bending stiffness of $2\times 10^{-23}$ N m$^2$, which is consistent with the experimental range of $10^{-24}{-}10^{-21}$ N m$^2$ [28, 43, 47, 57, 58]. The torque modulus for the rotation of the flagella is set to $|{\varvec{T}}|=350\ k_\mathrm{B}T =1450$ pN nm [28, 29], smaller than the stall torque of the rotary motor of flagella in *E. coli*, which is approximately 4500 pN nm [59]. The strength of the bond potential, $K_b$, and the excluded–volume interaction, $K_\mathrm{ex}$, are chosen as $K_b = K_\mathrm{ex} = 10^4 k_\mathrm{B}T/ a^2$.

The length, mass, and energy unit are chosen as to the length of a collision cell, *a*, the mass of a MPC particle, *m*, and the thermal energy, $k_\mathrm{B}T$. We mean number of particles in a collision cell is set to $\langle N_c \rangle = 10$, the rotation angle to $\alpha =130 ^{\circ }$, and the collision time to $h = 0.05 \sqrt{ma^2/(k_\mathrm{B}T)}$. These parameters yield a fluid viscosity of $\eta = 7.2 \sqrt{mk_bT}/a^2$. Newton’s equations of motion for the bacterial beads of mass $M=5m$ are integrated by the velocity-Verlet algorithm with the time step $\Delta t = 0.002\ \sqrt{ma^2/(k_\mathrm{B}T)}$. Periodic boundary conditions are applied with a cubic simulation box of side length $L_B=200 a^3$. We perform $10^6$ MPC steps for every realization. By this time, stationary bundles and a stationary swimming state are assumed.

Bacterial shape parameters The spectrum of *B. subtilis* morphologies is wide, owing to their ubiquity and large genomic diversity [23]. In particular, the ranges of body length and the number of flagella are wide. Moreover, prokaryotic flagella are far from being static objects, but are rather dynamic [60]. Several length-control and assembly models for the extracellular part of a flagellum have been suggested, but the underlying mechanism is still unclear [61]. The filament length is also affected by damage, especially when the swimming bacterium is exposed to external stresses, e.g., due to confinement. Flagella broken by mechanical shearing (e.g., during sample preparation) are able to regrow in similar Gram-negative species such as *E. coli* [60] and *S. enterica* [62, 63]. Other stresses can provoke irreversible damage to the flagella [64]. For our *B. subtilis*-like model bacterium, the cylindrical part of the body consists of 103 circles of diameter $d_b=9a$ with 101 central particles, and 18 rings for the two semi-spherical end caps, which yields the body length $l_b=62 a$. The thickness of a flagellum is determined by the range of the excluded–volume interactions between the flagella, i.e., $r_{\mathrm {ex}}^\mathrm{f}=0.25a$. With the relation $a \approx 0.1\, \upmu$m [28], these parameters correspond to the values listed in Table 1, well within experimentally determined ranges. *B. subtilis* cells of 4 $\upmu$m length synthesize up to 20 basal bodies [7, 65].

**Table 1:** Parameters for *B. subtilis*-like bacterial cells applied in the simulations and from experiments for the body length, $l_b$, diameter, $d_b$, the number of flagella, $N_f$, and their average length, $\langle L_f \rangle$

	Simulations	Exp. range	Refs.
$l_b$	6.2 $\upmu$m	4.0–6.5 $\upmu$m	[5, 66–68]
$d_b$	0.9 $\upmu$m	1.0 ± 0.1 $\upmu$m	[21, 67, 68]
$N_f$	14	5–25	[5, 19, 66, 67]
$\langle L_f \rangle$	5–7.5 $\upmu$m	$\lesssim \, 8.5$ $\upmu$m	[67]

The provided experimental values are taken from the listed references

### Flagellar surface distribution patterns

**Fig. 2:**
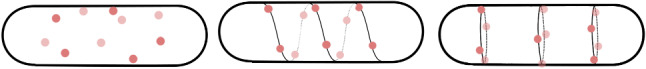
Sketch of flagellar anchoring patterns of peritrichous bacterial cell bodies: (left) random, (middle) helical, and (right) rings. Anchoring points are indicated as red dots. In *B. subtilis*, flagella are discouraged to grow at the poles, and the surface density of flagella is fore-aft symmetric [7]

We study three different anchoring patterns for the flagella: random, helical, and on rings (Fig. 2). No flagella are placed in the cap region.

(i) *Random anchoring*—For the random anchoring, an arbitrary bead of the cylindrical part of the body is selected. We consider 50 independent realizations for each of the six flagellum lengths.
(ii) *Helix*—The points along a helix are given by
  7 $$\begin{aligned} \varvec{r}_i = \left( r_b \cos \varphi _i, r_b \sin \varphi _i, i \Delta h \right) ^T \end{aligned}$$
  in Cartesian coordinates, with the cell body oriented along the *z*-axis of the Cartesian reference frame, and the body radius $r_b = d_b/2$. The angle $\varphi _i=2\pi \Delta (i-1) /30$ ($i \in \{1,N_f\}$), is the angle between the possible 30 anchoring points along a bead circle measured from a starting value We consider realizations with an odd number of body beads between subsequent anchoring sites, i.e., $\Delta = 3, \ldots , 29$) and the values $\Delta h/a= 6$ and 7. The first anchoring point is placed on the $3\mathrm{rd}$ ring of a cell’s cylindrical body part.
(iii) *Rings*—The most symmetric arrangement with the combination of 4–3–3–4 flagella placed on four rings is fore-aft symmetric with respect to the body’s main axis [7]. Specifically, the distances $\Delta _z =32$, 27, and 21 beads between the rings along the cylinder axis are considered. In addition, other combinations are studied, with a relative phase shift of the anchoring points on the various rings with respect to each other.

In every case, the flagellum lengths $L_f/a=50, 55, 60,65,$ 70,  and 75 are considered.

**Fig. 3:**
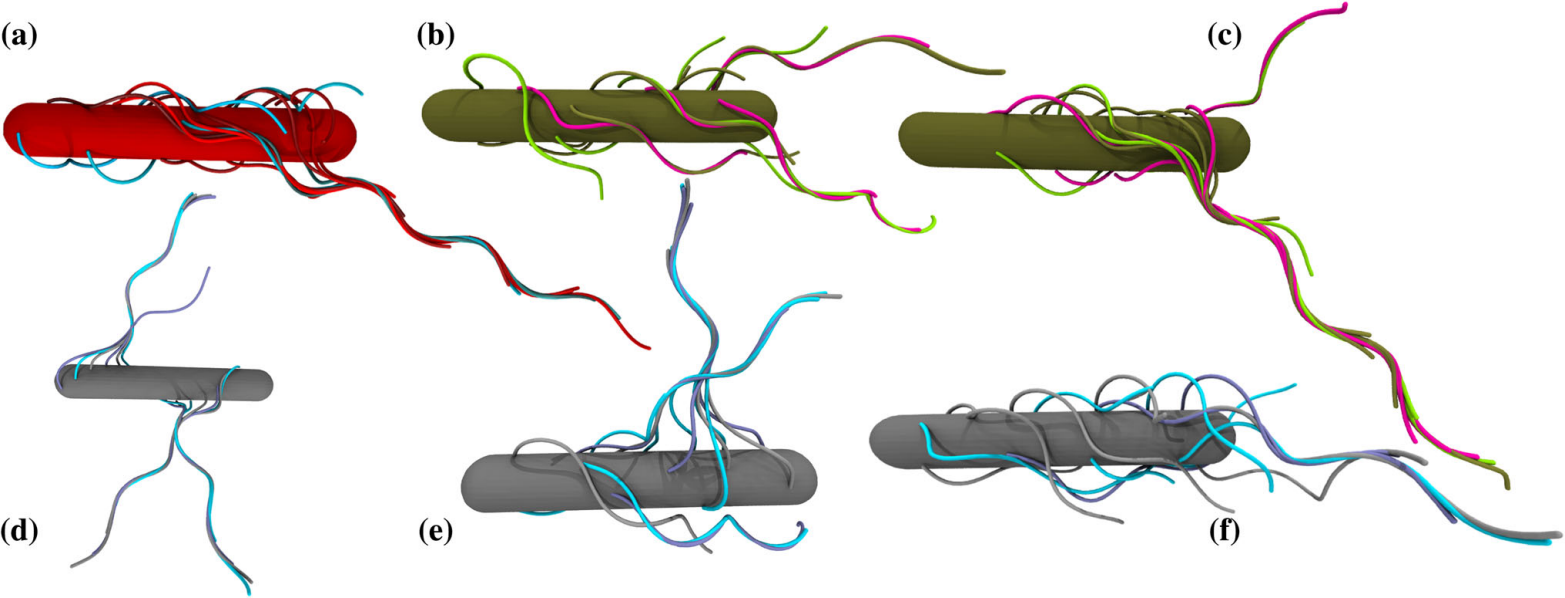
Classification and examples of bundle configurations. **a** Single bundle for helical anchoring. **b** Two rather symmetric V-shaped bundles for random anchoring. **c** Two asymmetric V-shaped bundles for helical anchoring. Cells (**b**) and (**c**) swim rather well. **d** “Other” configuration with three bundles pointing almost normally outward from the cell body for helical anchoring. **e** “Other” configuration with three bundles for random anchoring. **f** “Other” configuration of a hardly swimming cell with a single rudimentary bundle for flagellar anchoring on rings

## Flagellar bundling

Initially, flagella are anchored on the cell body according to the patterns described in Sect. 2.4 and the parameters of Table 1, with the individual flagella oriented perpendicular to the cell–body surface, i.e., pointing radially outward. By applying independent counterclockwise torques, $\varvec{T}$, the flagella start rotating, synchronize their rotation, and eventually form a bundle [37]. We define a bundle as composed of at least two flagella, which overlap by several backbone segments ($>2$) and do not simply touch or cross.

### Probability distribution of number of bundles

The emergent bundle configurations are classified into three categories: a single main bundle (1B) (Fig. 3a), two bundles (2B) of V-shaped structure (Fig. 3b, c)—in analogy of experimental findings [25]—, and “other” as illustrated in Fig. 3d–f. Under the term “other,” we collect realizations, where no bundle is formed, which means cells with individual flagella only, or multiple occurrences of structures with two intertwined flagella. In addition and more frequently, “other” realizations comprise structures, where bundles appear with an angle close to $90^{\circ }$ between the body and the bundle axes (Fig. 3d, e), i.e., the bundle points radially outward from the cylindrical body. The classification is certainly not unique and some of the configurations may change with time. Even more, various of the apparent “odd” bundle arrangements yield reasonably well-swimming cells. An example is shown in Fig. 3d for random anchoring. The cell moves while the body spins around a minor cylinder axis.

**Fig. 4:**
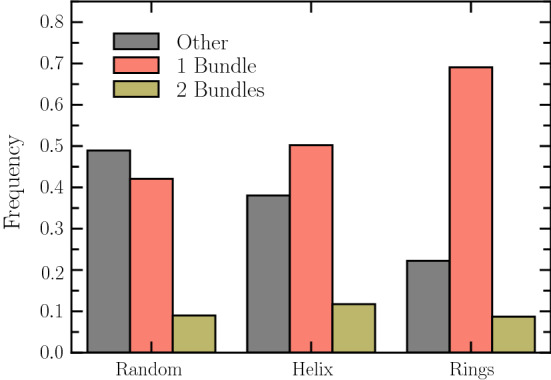
Relative occurrence of single-, two-bundle, and “other” configurations for the various anchoring schemes (random, helix, ring). Averages are presented for all considered flagellar lengths and anchoring patterns as described in Sect. 2.4

Figure 4 displays the average probability of single-, two-bundle, and “other” configurations for the considered anchoring patterns and all realizations. Evidently, the probability of a particular configuration depends on the anchoring pattern. “Other” structures are most likely for random anchoring, whereas for ring-like arrangements single bundles are most probable. A closer consideration of the latter structures shows that there are less flagella involved in the main bundle compared to other anchoring patterns, and they belong essentially to one ring at the rear of the cell. V-shaped structures of two bundles are nearly equally probable for all patterns, but are least likely for all patterns. Structures with more than two bundles in a V-shape configuration [25] are hardly observed in simulations within this model and the chosen parameter ranges. In general, we observe multiple bundles with two or more flagella most often wrapped around the cell body.

### Probability distribution of number of bundles: length dependence

**Fig. 5:**
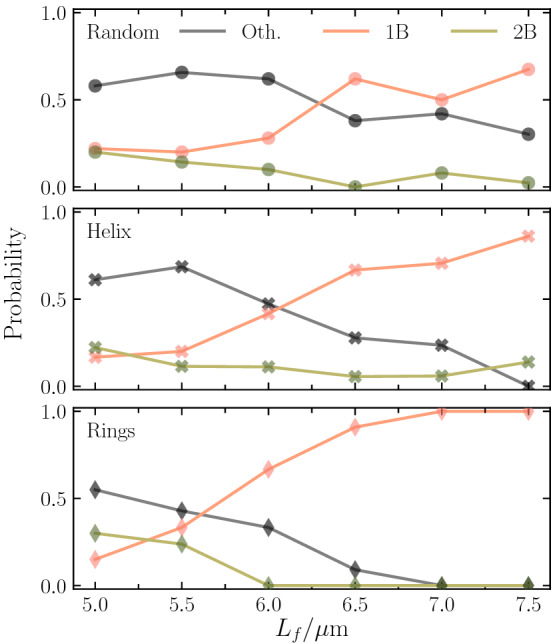
Dependence of the bundle probability (1B, 2B, “others”) on the flagellum length for (top) random, (middle) helical, and (bottom) ring-like arrangements of flagella. The lines are guides for the eye

The probability of forming a particular bundle depends on the flagellum length $L_f$, as shown in Fig. 5. The probability of “other” configurations is highest for shorter flagella and decreases with increasing $L_f$. On the contrary, cells equipped with flagella much longer than the body length form typically a single main bundle. Two bundles are most likely for short flagella and their probability decreases with increasing flagellum length.

The occurrence of the various configurations depends strongly on the anchoring pattern. For random anchoring, the rate for “others” drops slightly with increasing $L_f$, but is significant for all lengths. The two-bundle probability drops with increasing flagellar length to a rather small value. As a consequence, the likelihood for a single bundle increases and approaches a nearly constant value for $L_f/a \gtrsim 60$. A similar trend is obtained for the helical pattern, however, with a stronger drop of the probability of “other” configurations, but a small reminiscent probability for two bundles and a high probability for a single bundle. The most pronounced length dependence follows for ring patterns, where the “other” and two-bundle probability drops to zero with increasing $L_f$, and only cells forming a single main bundle are present. For rings, a major change occurs when the flagellum length exceeds the body length, i.e., for $L_f \gtrsim 6\, \upmu$m. Here, bundles are preferentially formed at the rear part of the cell body—rear in the sense of the finally formed bundle at this part, which is governed by the counterclockwise rotation of the flagella—and include mainly flagella from the ring at the rear part.

**Fig. 6:**
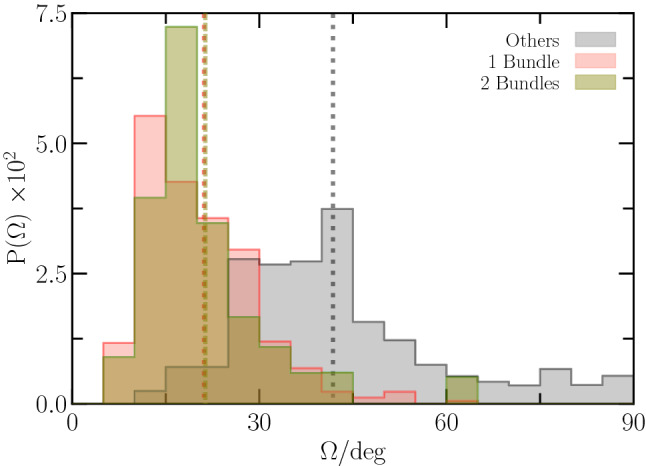
Distribution function, $P(\Omega )$, of the wobbling angle, $\varOmega$, for single-, two-bundle, and “other” configurations. The area under the individual curves is unity

**Fig. 7:**
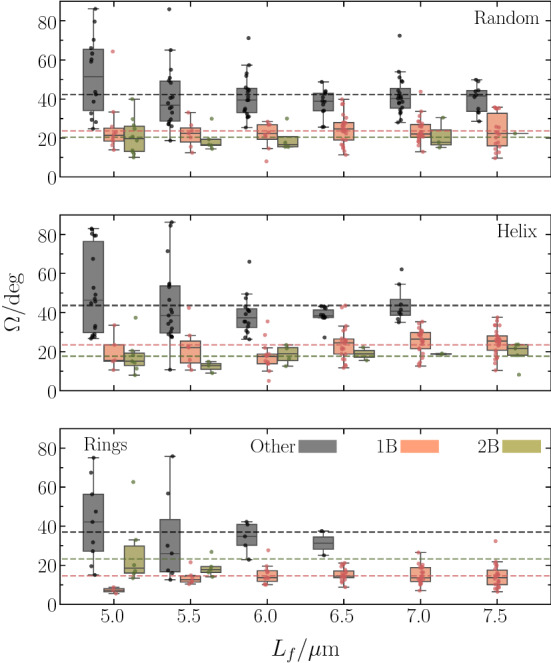
Box plot of the dependence of the average wobbling angle on the anchoring pattern for single-, two-bundle, and “other” configurations [69]. Bullets represent the individual realizations. The lines indicate averages over the various lengths. The gray and green boxes for a particular length are shifted with respect to the red one for better visibility

### Wobbling angle

Flagellar bundles are typically inclined with the cell–body cylinder axis (Fig. 3), which implies cell wobbling, i.e., precession of the cell–body major axis around the swimming direction [30, 47, 70–73]. Figure 6 shows the dependence of the wobbling angle, $\varOmega$, on the swimmer configuration, where $\varOmega$ follows from the product $\langle \varvec{e}_b \cdot \varvec{e}_s \rangle = \cos \varOmega$ of the unit vectors of the body main axis, $\varvec{e}_b$, and the swimming direction, $\varvec{e}_s$. The average is taken over a cell’s trajectory. The probability distribution functions for single- and two-bundle configurations are rather similar, whereas that for “other” configurations is shifted to larger $\varOmega$. Noteworthy, the distribution of “other” configurations exhibits a long tail with rather large angles. The mean values $\bar{\varOmega }$ for the various anchoring patterns are $\bar{\varOmega } = 21^{o}$ (1B, 2B) and $42^{o}$ (“other”). The single-bundle and two-bundle configurations yield the same average, whereas $\bar{\varOmega }$ of “other” is about twice larger. This indicates a strong inclination of the propulsion direction with the body axis.

Figure 7 displays the dependence of the wobbling angle on the flagellum length for the various anchoring patterns. There is only a weak dependence of $\varOmega$ on $L_f$ for all bundles and anchoring patterns. However, the variance of $\varOmega$ for “other” configurations is typically significantly larger than that of single- and two-bundle configurations.

The wobbling angles of the considered long cell with a rather large number of flagella are larger than those of the *E. coli* cells considered experimentally [72, 73] and in simulations [30]. The shorter cell body of *E. coli* favors bundle formation at the rear part of the cell and a more parallel alignment of cell body and flagellar bundle, and correspondingly weak wobbling [29]. Specifically for short flagella, this is not the case for the longer cells and bundles are correspondingly more oblique with respect to the body main axis, implying larger wobbling angles.

**Fig. 8:**
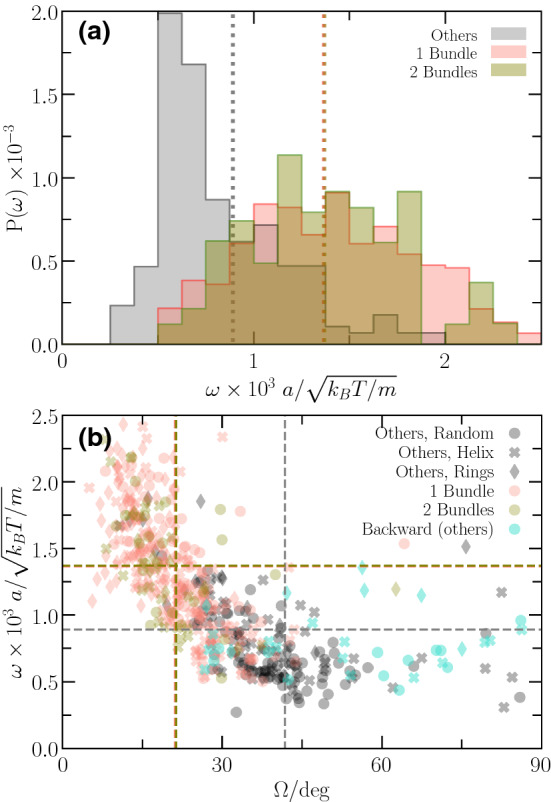
**a** Probability distribution, $P(\omega )$, of the wobbling frequency, $\omega$, for single-, two-bundle, and “other” configurations. The distribution functions are normalized such that the area is unity. **b** Scatter plot of the wobbling frequency and the wobbling angle. The different symbols and colors indicate the various anchoring patterns and number of bundles, respectively. In particular, cyan symbols correspond to backward swimming realizations. The dashed lines indicate average values

### Wobbling frequency

The distribution function of the wobbling frequency $\omega = 2\pi /\tau$, where $\tau$ is the time for a full rotation of the cell body around the swimming direction during precession, is displayed in Fig. 8a. Single- and two-bundle configurations exhibit a rather broad distribution of frequencies, whereas $P(\omega )$ of “other” configurations shows a narrow peak in the vicinity of $\omega /\sqrt{m a^2/(k_\mathrm{B}T)} \approx 6 \times 10^{-4}$ with a long tail toward larger $\omega$.

The distribution function of the two-bundle configurations is rather coarse, because the number of such configurations is small. Here, more realizations are necessary to achieve a smooth function.

The mean values $\bar{\omega }$ for the various anchoring patterns are $\bar{\omega }/\sqrt{m a^2/(k_\mathrm{B}T)} \approx 9\times 10^{-4}$ (“other”) and $1.4 \times 10^{-3}$ (1B, 2B). The single- and two-bundle swimmers show a 1.5 times higher $\omega$, which is related to their smaller wobbling angle as reflected in Fig. 8b.

Figure 8b indicates a strong dependence of the wobbling frequency on the wobbling angle. In general, $\omega$ decreases with increasing $\varOmega$, with little difference between single- and two-bundle configurations. Consistent with Figs. 6 and 8a, “other” configurations show larger $\varOmega$ and lower frequencies. Noteworthy, we find various bundle arrangements, where cells swim backward, independent of the anchoring pattern (cyan symbols in Fig. 8b), where backward means in the direction of a bundle at the rear part of a cell. So far, we don’t have a satisfactory explanation of the phenomenon, but the effect should be related to the wrapping around the cell body.

**Fig. 9:**
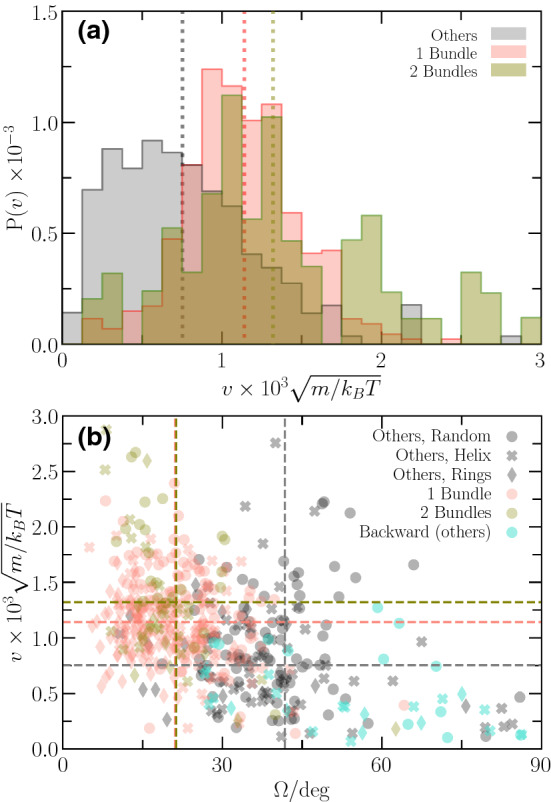
**a** Probability distribution, *P*(*v*), of the magnitude of the swimming speed, *v*, for single-, two-bundle, and “other” configurations. All configurations are included, independent of the anchoring pattern and the length of the flagella. A distribution function is normalized such that the area is unity. **b** Scatter plot of the swimming speed and the wobbling angle. The different symbols and colors indicate the various anchoring patterns and number of bundles, respectively. In particular, cyan symbols correspond to backward swimming realizations. The dashed lines indicate average values

**Fig. 10:**
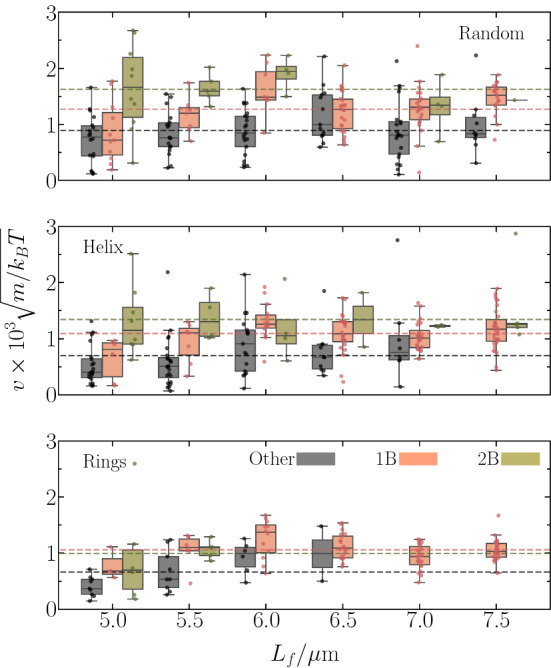
Box plot of the dependence of the swimming speed on the anchoring pattern for single-, two-bundle, and “other” configurations [69]. Bullets represent the individual realizations. The lines indicate averages over the various lengths. The gray and green boxes for a particular length are shifted with respect to the red one for better visibility

## Cell dynamics

### Swimming speed

The distribution function, *P*(*v*), of the swimming speed, *v*, is displayed in Fig. 9a, where *v* is calculated from the fairly linear displacement of a cell’s center-of-mass after stationary swimming is assumed, where the displacement can reach several body lengths; explicitly, *v* is the ratio of that displacement and the corresponding time interval. Note that a few no-swimming realizations are disregarded in this analysis. Since the cells swim ballistically on average (Fig. 12), the displacement grows essentially linearly with time. The swimming speed depends moderately on the kind of emerging bundle (Fig. 9a). The “other” configurations exhibit a broad peak with a maximum at $v/\sqrt{k_\mathrm{B}T/m} \approx 5.5 \times 10^{-4}$ and a long tail toward larger velocities. The distribution function for single-bundle configurations is more symmetric, but similarly broad. The probability distribution of two-bundle configurations is very broad and shows several groups; the latter is most likely a consequence of the rare appearance of such configurations. Correspondingly, the average swimming speed, $\bar{v}/\sqrt{k_\mathrm{B}T/m} \approx 1.3 \times 10^{-3}$, is largest for two-bundle configurations and reduces to $\bar{v}/\sqrt{k_\mathrm{B}T/m} \approx 1.1 \times 10^{-3}$ and $8 \times 10^{-4}$ for single-bundle and “other” configurations, respectively. Hence, cells with two-bundles swim approximately $60\%$ faster than cells with “other” configurations, but show little dependence on the anchoring pattern.

As shown in Fig. 9b, the swimming speed decreases with increasing wobbling angle, in particular for single- and two-bundle configurations. Overall, the backward swimming realizations show the smallest average swimming speed.

### Swimming speed as function of flagellum length

In general, we find a weak dependence of the swimming speed on the flagellum length, as displayed in Fig. 10. Cells with shorter flagella ($L_f < 5.5 \,\upmu {\mathrm {m}}$) swim slower by trend, which is most pronounced for helical and ring arrangements, and “other” and single-bundle configurations. With increasing flagellar length, the effect disappears and the swimming speed becomes independent of $L_f$. We assume that a stronger (frictional) interaction of the short flagella with the cell body leads to a less effective propulsion and, hence, to a smaller swimming speed.

Unexpectedly, the swimming speed for the ring pattern is slowest. At a first glance, we could have expected that the flagella of the rear ring would form a bundle nearly aligned with the cell body resulting in efficient propulsion. However, this seems not to lead to efficient swimming or is counteracted by the interactions of other flagella with the body. In general, the largest swimming speeds for single-, two-bundle, and “other” configurations are achieved for random anchoring.

**Fig. 11:**
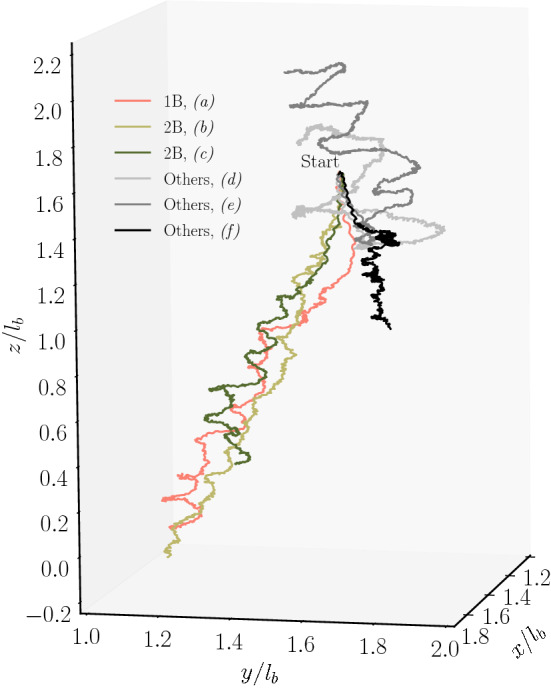
Trajectories of the body center of mass for the flagellar arrangements displayed in Fig. 3. Initially, all cells are localized at “Start.” The “oscillation” amplitudes, which are significantly smaller than the body length, reflect the extent of wobbling

### Mean-squared displacement of the cell body

The different extent of bundling leads to a significant disparity in the cell dynamics. This is reflected in the trajectories displayed in Fig. 11. The cell with the symmetric two bundles shows the smoothest, most straight trajectory, and the largest displacement. Similarly, the cell with the single major bundle swims rather straight and fast. Cells with “other” configurations swim along helical-like trajectories, which can show rather large amplitudes reflecting the extent of wobbling.

**Fig. 12:**
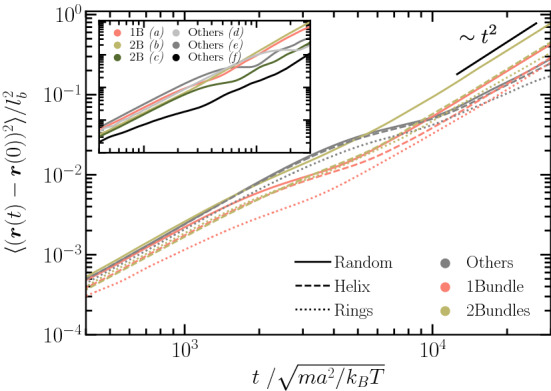
Mean-square displacement of the center of mass of the cell body for the various anchoring patterns and bundle configurations. Inset: mean-square displacements for the particular structures presented in Fig. 3

Figure 12 presents the mean-square displacement (MSD) of the cell–body center of mass for the various anchoring patterns and bundle configurations. The time interval of bundle formation at the beginning of the simulation is excluded from the analysis. MSDs are calculated from stationary states, where only further minor bundle adjustments occur. For short times, $t/\sqrt{ma^2/(k_\mathrm{B}T)} \approx 10^{2}$, most of the cells exhibit a very similar ballistic motion. The plateau-like regimes for longer times are a consequence of the cells’ wobbling motion; here, the MSD corresponds to about half a period of the oscillations as, e.g., visible in Fig. 11. For even longer times, again a (nearly) ballistic motion is obtained [3], but with a wider range of MSDs, reflecting the disparity of swimming speeds of the different bundle configurations and anchoring patterns. Similar to the swimming speed displayed in Figs. 9 and 10, the MSD is largest for two-bundle configurations for each of the anchoring patterns. Most remarkably, however, is the fact that random anchoring in any case yields the largest MSD. Overall, the majority of the considered realizations exhibit swimming at longer times, i.e., they move ballistically. An exception is “other” configurations for ring anchoring (gray dotted line). Here, the MSD is not increasing quadratically with *t* for times $t/\sqrt{ma^2/(k_\mathrm{B}T)} > 2 \times 10^{4}$, reflecting their reduced swimming abilities. On much longer time scales, longer than the inverse of the rotational diffusion coefficient of a cell, the MSD will crossover to an active diffusive motion. However, rotational diffusion of even the cell body around its minor axis is very slow and, hence, the diffusive regime will be hardly reached in our simulations. The inset of Fig. 12 shows MSDs for the conformations displayed in Figs. 3 and 11. The disparity of the various curves emphasizes the strong dependence of the cell’s swimming ability on the particular flagellar organization.

## Summary and conclusions

We have performed intensive simulations to study bundle formation and the swimming speed of *B. subtilis*-like bacteria with various flagellar anchoring patterns, namely random, helical, and anchoring on rings. The various flagellar arrangements reveal distinct differences in terms of the formation of flagellar bundles and the swimming speed. By our definition of a bundle consisting of at least two flagella, we find preferentially cell conformations with single and two bundles of V-shape. In addition, there are cell configurations with many individual flagella and/or further bundled flagella, which we classify as “other.” In experiments, up to four bundles for *B. subtilis* have been reported [25]. We can think of several reasons for the discrepancy with our simulations. First, the flagellum length and number in experiment and simulations could be different. Second, the flagella in experiments are not of uniform length; length dispersion, specifically, the presence of very long or short flagella may promote formation of multiple bundles. Third, the flagella in biological systems grow with time, i.e., the flagellar length varies with time. The effect of polydispersity in flagellum length on flagellar bundling is unclear and remains to be elucidated. Fourth, identification of a bundle, rather than individual flagella or groups of nearby (non-bundled) flagella, might be difficult in an experiment. In addition, we characterize peculiar structures as “other,” where a cell even with several bundles may hardly swim, swim backward, or might even spin around a minor axis with bundles essentially normal to the cell–body major axis. Naturally, the number of bundles and their orientation with respect to the cell body can change with time, an aspect not taken into account in the present study, where the bundling state has been classified after a cell reached a stationary swimming sate.

In terms of anchoring patterns, ring arrangements show a preference for single bundles, in particular when the flagellum length exceeds the body length. In cells with randomly anchored flagella, the probability of “other” configurations slightly dominates over single-bundle configurations. In any case, the likelihood for V-shaped bundles is significantly smaller.

Single- and two-bundle configurations imply similar average wobbling angles, which are significantly smaller than that of “other” configurations. The latter is a consequence of the often rather oblique orientation of the flagella with respect to the body axis, independent of the anchoring pattern. This leads to lower wobbling frequencies of the “other” configurations. Most important, the swimming speed of “other” configurations is also smaller than those of single-and two-bundle configurations. The largest swimming speed follows for two-bundle configurations. Interestingly, the highest average swimming speeds are obtained for random anchoring, for all bundle structures. The relation between the swimming speed and the wobbling angle (Fig. 9b) is in qualitative agreement with experimental results [25]. In both studies, *v* decreases with increasing $\varOmega$. However, the most probable wobbling angle is smaller in experiments compared to our value. This is, at least partially, related to the pronounced V-shape structures in experiments. As pointed out, a V-shape bundle arrangements exhibit small wobbling angles in simulations.

Our studies strongly suggest that there is no advantage of a regular flagellum anchoring pattern in terms of swimming speed. In contrast, random anchoring allows to form single- and two-bundle configurations resulting in faster cell swimming than regular anchoring patterns. This faster motion seems to be related (to some extent) to a larger wobbling angle, which suggests that a narrow distribution of flagella close to the cell body is of disadvantage for swimming by a more pronounced frictional interaction between flagella and cell body.
